# Trans‐Fatty Acids (TFA) Induced Vascular Injury Through the Regulation of the Sirt1‐Ppargc1a‐Nfe2l2 Signaling Pathway in Male Rats

**DOI:** 10.1002/fsn3.70975

**Published:** 2025-09-26

**Authors:** Huanting Pei, Ran Li, Yadong Zhang, Xiaoya Sun, Siqi Zhu, Simeng Qiao, Chongyue Zhang, Xiaolong Zhang, Jingyi Ren, Yuxia Ma

**Affiliations:** ^1^ Department of Nutrition and Food Hygiene, School of Public Health Hebei Medical University, Hebei Key Laboratory of Environment and Human Health Shijiazhuang China; ^2^ Tianjin Hospital Tianjin China

**Keywords:** apoptosis, Sirt1, trans‐fatty acids, vascular injury

## Abstract

Dietary trans‐fatty acids (TFA) elevate cardiovascular disease (CVD) risk by driving vascular injury, yet the mechanisms underlying this effect remain largely unexplored. Eighteen male Sprague–Dawley (SD) rats were randomly assigned to three groups: control group, a low‐dose TFA diet group, and a high‐dose TFA diet group. During the 12‐week experiment, body weight, food intake, and blood pressures were monitored. Post‐experiment, serum triglycerides (TG), total cholesterol (TC), high‐density lipoprotein cholesterol (HDL‐C), and low‐density lipoprotein cholesterol (LDL‐C) were quantified using assay kits. Network pharmacology, molecular docking, and western blotting were employed to predict and validate the toxicological mechanisms underlying TFA‐induced vascular injury. TFA significantly induced mitochondrial damage in the vascular tissues of rats. TFA consumption increased serum TC, TG, and LDL‐C levels while reducing HDL‐C levels in rats. Network pharmacology analysis revealed significant enrichment in apoptotic pathways, highlighting the pivotal roles of Sirt1, Ppargc1a, and Nfe2l2 in the mechanistic network underlying TFA‐induced vascular injury. Furthermore, molecular docking and western blotting analyses validated substantial changes in protein expression linked to apoptosis and the Sirt1‐mediated signaling pathway. TFA induces mitochondrial damage, elevating reactive oxygen species levels and triggering vascular apoptosis in rats. This process is mediated by modulation of the Sirt1/Ppargc1a/Nfe2l2 signaling pathway. These findings clarify a critical mechanism of TFA‐induced vascular injury, highlighting dietary TFA as a modifiable risk factor for cardiovascular health.

## Graphical abstract

This study firstly suggested that trans‐fatty acids (TFA) can induce vascular apoptosis. Network toxicology and molecular docking were used to explore mechanisms. This study identified Sirt1 as key in apoptosis caused by TFA.

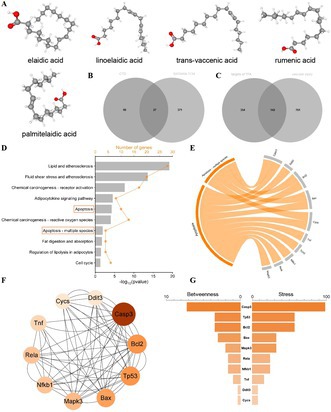

## 1 Introduction

Trans‐fatty acids (TFA) are a distinct class of unsaturated fatty acids (UFA) characterized by the presence of one or more trans carbon–carbon double bonds (Magtanong et al. 2019). In humans, the enzymes responsible for fatty acid desaturation exclusively introduce cis double bonds into fatty acids. As a result, enzymatically synthesized UFA contain only cis double bonds, hereafter referred to as cis‐fatty acids (CFA), whereas TFA are exclusively derived from dietary sources (Micha and Mozaffarian 2008). According to their dietary sources, TFA can be classified into 2 categories: industrially produced TFA (iTFA) and natural TFA (ruminant TFA, rTFA) (Jasińska‐Melon et al. 2022). iTFA, including elaidic acid (18:1 trans‐9) and linoelaidic acid (18:2 trans‐9, trans‐12), are primarily generated as byproducts during food manufacturing processes, particularly during the partial hydrogenation of edible oils containing CFA. These compounds are commonly found in processed foods, especially in snacks and fast food items (Pipoyan et al. 2022). By contrast, rTFA, including trans‐vaccenic acid (18:1 trans‐11), rumenic acid (18:2 cis‐9, trans‐11), and palmitelaidic acid (16:1 trans‐9), are predominantly generated through bacterial biohydrogenation of CFA within the rumen of ruminants, such as cows and sheep (de Brito et al. 2021). These rTFA are present in meat products in daily life (Hanula et al. 2022). Accumulating epidemiological evidence has demonstrated that TFA consumption is associated with an increased risk of several diseases, including cardiovascular diseases (CVDs) (Gao et al. 2022), diabetes (Mi et al. 2022), systemic inflammation (Kikut et al. 2021), and neurodegenerative diseases (NDs) (Morris et al. 2003). It has been estimated that a 1% reduction in the consumption of iTFA could prevent approximately 72,000 deaths annually related to CVDs (Mozaffarian and Stampfer 2010). It is worth noting that one common feature in the above‐mentioned diseases is vascular injury. At present, there is a small number of studies suggesting that TFA may have the potential to induce vascular injury (Harvey et al. 2008; de Roos et al. 2003). However, the specific toxicological mechanisms underlying this effect remain poorly understood and require further investigation.

Silent information regulator 1 (Sirt1) is a critical mediator of intracellular protective mechanisms in response to cellular damage (DiNicolantonio et al. 2022; Qiu et al. 2022). In addition to its well‐documented antioxidant properties, Sirt1 is distinguished by its antiapoptotic properties (Cui et al. 2022). It promotes the activation of nuclear factor erythroid 2‐related factor 2 (Nfe2l2), thereby upregulating the expression of protective enzymes, such as glutathione peroxidase (GPx) and superoxide dismutase (SOD), through the antioxidant response element (Dang et al. 2022; Arioz et al. 2019). Emerging evidence highlights the significant role of Sirt1 in maintaining mitochondrial homeostasis and mitigating cardiac ischemia–reperfusion injury (Zhong et al. 2019). Moreover, Sirt1 can also increase the activity of peroxisome proliferator‐activated receptor gamma coactivator 1‐alpha (Ppargc1a), which plays a crucial role in maintaining mitochondrial function and regulating energy metabolism (Mei et al. 2020; Yuan et al. 2012). The deacetylation of Ppargc1a by Sirt1 promotes mitochondrial biogenesis and exerts antiapoptotic effects by modulating the Bax/Bcl‐2 ratio (Mu et al. 2023). Despite these insights, research exploring the relationship between fatty acids and Sirt1 remains limited. A few studies have demonstrated that omega‐3 polyunsaturated fatty acids (PUFA) can upregulate Sirt1 expression, thereby attenuating inflammatory responses (Inoue et al. 2017; Wang et al. 2017). However, the regulatory effects of TFA on Sirt1 have not been investigated, leaving a significant gap in understanding the interplay between dietary fatty acids and Sirt1‐mediated cellular protection.

In this study, we employed a randomized experimental design in which rats were allocated to distinct dietary groups, each administered varying concentrations of TFA. The primary objective of this investigation was to elucidate the potential role of TFA in inducing vascular apoptosis through the modulation of Sirt1 signaling pathways.

## 2 Materials and Methods

### 2.1 Materials and Reagents

Trans‐fatty acids were obtained from hydrogenated palm oil containing a high TFA concentration (46%), provided by a commercial oil and fat supplier. The experimental diets were formulated and prepared by Beijing Nuokang Source Biotechnology Co. Ltd. The detailed composition of the feed for each dietary group is summarized in Table 1. Protein quantification was performed using the Lowry method, and the quantified proteins were subsequently subjected to western blot analysis. The following primary antibodies were employed in the study: anti‐rabbit Bcl2 (Signalway Antibody, China), anti‐rabbit Bax (Cell Signaling Technology, USA), anti‐rabbit Ppargc1a (Affinity, China), anti‐rabbit Casp3 (GeneTex, USA), anti‐rabbit Cleaved Casp3 (Arigo Biolaboratories, China), anti‐rabbit Nfe2l2 (Medical & Biological Laboratories, China), anti‐rabbit Sirt1 (OriGene, USA), and anti‐rabbit β‐actin (Abcam, China). The secondary antibody, goat anti‐rabbit IgG‐HRP (horseradish peroxidase), was procured from Santa Cruz Biotechnology (USA).

**TABLE 1:** Nutrient content of the feed.

Ingredient	ND (g)	LTD (g)	HTD (g)
Carbohydrate	56.37	56.37	56.37
Fat	4.11	3.29	0.80
Hydrogenated vegetable oil	0.00	0.82	3.31
Protein	19.98	19.98	19.98
Coarse ash	5.90	5.90	5.90
Moisture	9.50	9.50	9.50
Calcium	1.18	1.18	1.18
Phosphorus	0.88	0.88	0.88
Cellulose	2.06	2.06	2.06
Total	100.00	100.00	100.00

### 2.2 Animal Treatment

Eighteen male Sprague–Dawley (SD) rats (body weight: 180 ± 20 g) were purchased from Beijing Vital River Laboratory Animal Technology Co. Ltd. Animals were acclimatized for 1 week under standard laboratory conditions (temperature: 20°C–24°C, humidity: 50%–70%, 12‐h light–dark cycle) prior to experimentation. Rats were then randomly allocated into three groups (*n* = 6 per group): (i) Control group (ND, normal diet without TFA), (ii) Low‐dose TFA diet group (LTD, 1% of the total energy as TFA), and (iii) High‐dose TFA diet group (HTD, 4% of the total energy as TFA). Body weight and food consumption were measured weekly. Systolic and diastolic blood pressure were recorded at 6‐week intervals. After 12 weeks of dietary intervention, rats were euthanized via intraperitoneal injection of sodium pentobarbital (50 mg/kg body weight). Abdominal aorta tissues and serum samples were subsequently collected for analysis.

### 2.3 Histological Analysis

Following extraction, vascular tissues were rinsed with ice‐cold phosphate‐buffered saline (PBS) and fixed in 4% paraformaldehyde for 24 h at 4°C before paraffin embedding. Paraffin‐embedded sections (4 μm thickness) were prepared for histopathological analysis. Hematoxylin and eosin (H&E) staining was performed to evaluate inflammatory infiltration, whereas Weigert staining was utilized to assess collagen deposition and elastic fiber integrity (Ren et al. 2023; Huo et al. 2024). Histopathological scoring of inflammation and fibrosis was conducted using established criteria. Elastic fiber morphology and distribution were quantitatively analyzed across experimental groups using the image analysis software (e.g., ImageJ) (Huo et al. 2024).

### 2.4 Measurement of ROS Generation in Vascular

Intracellular reactive oxygen species (ROS) levels were measured using the fluorescent probe 2′,7′‐dichlorodihydrofluorescein diacetate (DCFH‐DA). Fresh, unfixed abdominal aorta segments were sectioned into tissue slices and incubated with 10 mmol/L DCFH‐DA in a light‐protected chamber at 37°C for 30 min. ROS levels were subsequently quantified using fluorescence microscopy.

### 2.5 Electron Microscope Observations

Upon euthanasia, tissues were immediately collected and washed with physiological saline. A segment of the abdominal aorta, approximately 2 mm in length, was rapidly fixed in 4% glutaraldehyde. Following fixation, the tissues were sectioned into thin slices and subjected to histological examination using electron microscopy. Ultrastructural alterations in the mitochondria were observed and documented for subsequent analysis.

### 2.6 Serum Biochemical Tests

For biochemical analyses, plasma and serum samples were isolated and processed to evaluate relevant biochemical parameters. Serum levels of triglycerides (TG), total cholesterol (TC), high‐density lipoprotein cholesterol (HDL‐C), and low‐density lipoprotein cholesterol (LDL‐C) were measured using a fully automated biochemical analyzer. All procedures were performed at the First Affiliated Hospital of Hebei Medical University.

### 2.7 Network Pharmacology Prediction

Potential targets of TFA were predicted utilizing the Comparative Toxicogenomics Database (CTD; https://ctdbase.org/) and the Bioinformatics Annotation Database for Molecular Mechanism of Traditional Chinese Medicine (BATMAN‐TCM; http://bionet.ncpsb.org.cn/batman‐tcm/#/home). To identify vascular injury‐related targets, the keyword “vascular injury” was queried in the Genecards database (https://www.genecards.org/), and the top 10% of genes with the highest relevance scores were selected for further analysis. The intersection of TFA targets and vascular injury‐related targets was considered potential targets of TFA‐induced vascular injury. Kyoto Encyclopedia of Genes and Genomes (KEGG) pathway enrichment analysis was subsequently performed on the identified targets using the DAVID database (https://davidbioinformatics.nih.gov/). Results with a *p* value < 0.05 were deemed statistically significant and visualized using an online bioinformatics platform (https://www.bioinformatics.com.cn/). Protein–protein interaction (PPI) networks were constructed using the STRING database (https://cn.string‐db.org/) and analyzed using the Cytoscape software (version 3.9.1). Hub proteins were identified by applying two topological algorithms (Betweenness and Stress), and the top 10 nodes with the highest scores from each algorithm were selected for further evaluation.

### 2.8 Docking Analysis

The three‐dimensional structure of Sirt1 was obtained from the AlphaFold database via UniProt (https://www.uniprot.org/). The PubChem CID (Compound Identifier) numbers of the molecules were retrieved from the PubChem database (https://pubchem.ncbi.nlm.nih.gov/). Molecular docking simulations were conducted using an online computational platform (https://www.home‐for‐researchers.com/#/). The resulting docking complexes were visualized and analyzed using the PyMOL software (version 2.5.2). The binding affinity of TFA to Sirt1 was evaluated based on binding energy, expressed in kcal/mol.

### 2.9 Western Blotting

Vascular tissue samples were homogenized in RIPA buffer supplemented with protease inhibitors and centrifuged at 12,000 rpm and 4°C to isolate the protein supernatant. The supernatant was denatured by heating in 5× SDS loading buffer at 95°C for 5 min. Proteins were separated by 8%–12% SDS‐PAGE and electrophoretically transferred onto PVDF membranes. The membranes were blocked with 5% skimmed milk for 2 h at room temperature, then cut into strips and incubated with primary antibodies at 4°C overnight. After incubation, the membranes were washed three times with TBST (10 min per wash) and incubated with the corresponding horseradish peroxidase (HRP)‐conjugated secondary antibodies for 1 h at room temperature. Following another three TBST washes, protein bands were visualized using an enhanced chemiluminescence (ECL) detection system. Band intensities were quantified using the ImageJ software (version 1.53), and protein expression levels were normalized to β‐actin as an internal control.

### 2.10 Statistical Analysis

Statistical analyses were performed using the Statistical Package for Social Sciences (SPSS, version 20.0) and GraphPad Prism (version 6.0). Data are expressed as mean ± standard deviation (SD) for all experiments. Differences among treatment groups were assessed using one‐way analysis of variance (ANOVA), with statistical significance defined as *p* < 0.05.

## 3 Result

### 3.1 TFA Promotes Mitochondria Damage in Rat Vascular Tissues

**FIGURE 1:**
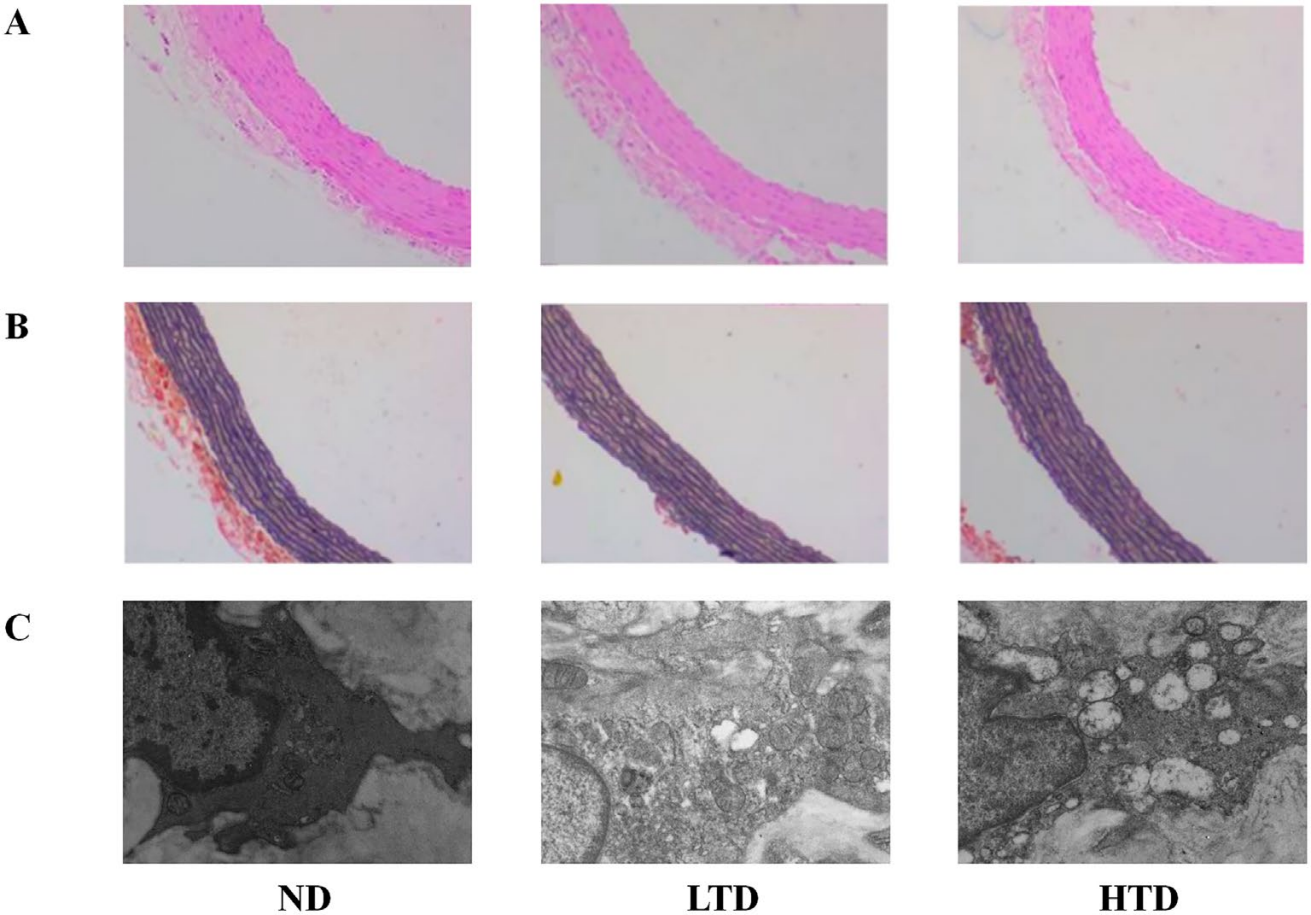
Histological aspects of vascular tissue sections from different groups of rats. (A) Hematoxylin and eosin (H&E) staining of cardiovascular sections (200×). (B) Weigert staining of cardiovascular sections (200×). (C) Electron microscopy of the mitochondrial morphology of blood vessels. ND, normal diet; LTD, 1% trans‐fatty acids diet; HTD, 4% trans‐fatty acids diet.

To evaluate the extent of vascular injury induced by varying levels of TFA, both histological and ultrastructural analyses were performed. Hematoxylin and eosin staining revealed that vascular tissue cells in the ND, LTD and HTD groups maintained normal morphology and were orderly arranged, with no significant differences observed among the three groups (Figure 1A). To determine whether TFA levels affected elastic fibers in the vascular tissue, Weigert staining was conducted. The results indicated no significant disruption of elastic fibers across the three groups (Figure 1B). However, electron microscopy analysis demonstrated that, compared with the control group, mitochondrial swelling and vacuolization were markedly increased in both the LTD and HTD groups (Figure 1C).

### 3.2 Effect of TFA on Body Weight, Food Intake, and Blood Pressures

**FIGURE 2:**
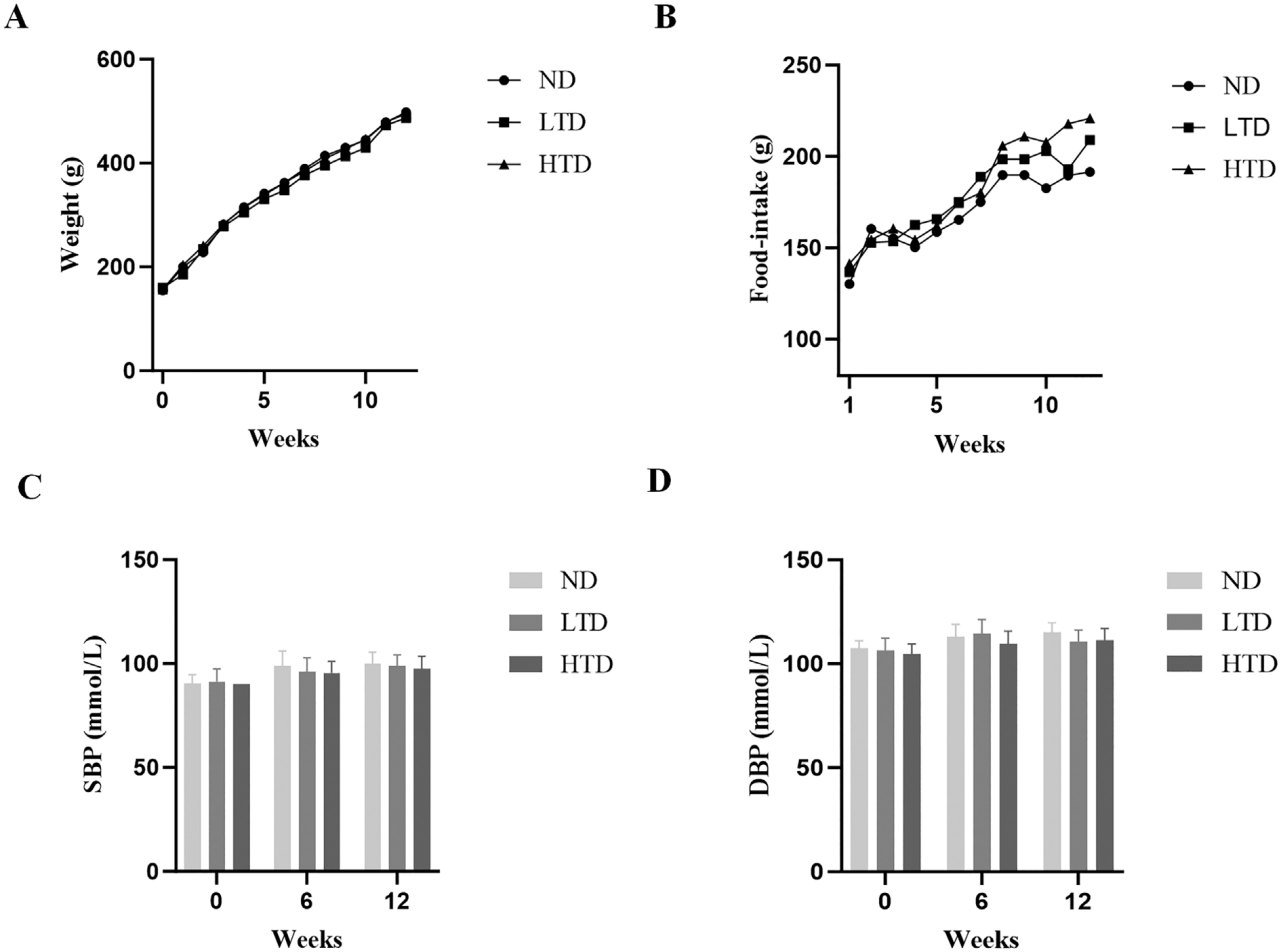
Effects of trans‐fatty acids on body weight, food intake, and blood pressures in different groups. (A) Weekly cumulative weight gain of rats. (B) Weekly food intake of rats. (C, D) Systolic blood pressure (SBP) and diastolic blood pressure (DBP) of the three groups of rats at 0, 6, and 12 weeks. Results are shown as mean ± SD (*n* = 6). ND, normal diet; LTD, 1% trans‐fatty acids diet; HTD, 4% trans‐fatty acids diet.

To evaluate the impact of TFA on physiological parameters, body weight, food intake and blood pressure were systematically monitored throughout the experimental period. The results are summarized in Figure 2. Both body weight and food intake exhibited a gradual increase with age in all groups, consistent with normal physiological growth. However, no statistically significant differences were observed between the TFA‐treated groups and the control group (Figure 2A,B). Similarly, blood pressure measurements, including systolic and diastolic pressures, remained comparable across all groups, with no significant variations attributable to TFA administration (Figure 2C,D). In conclusion, these findings indicate that TFA exposure did not induce significant alterations in the assessed physiological indices, including body weight, food intake, and blood pressure under the experimental conditions employed in this study.

### 3.3 Effect of TFA on Serum Lipid Indexes

**FIGURE 3:**
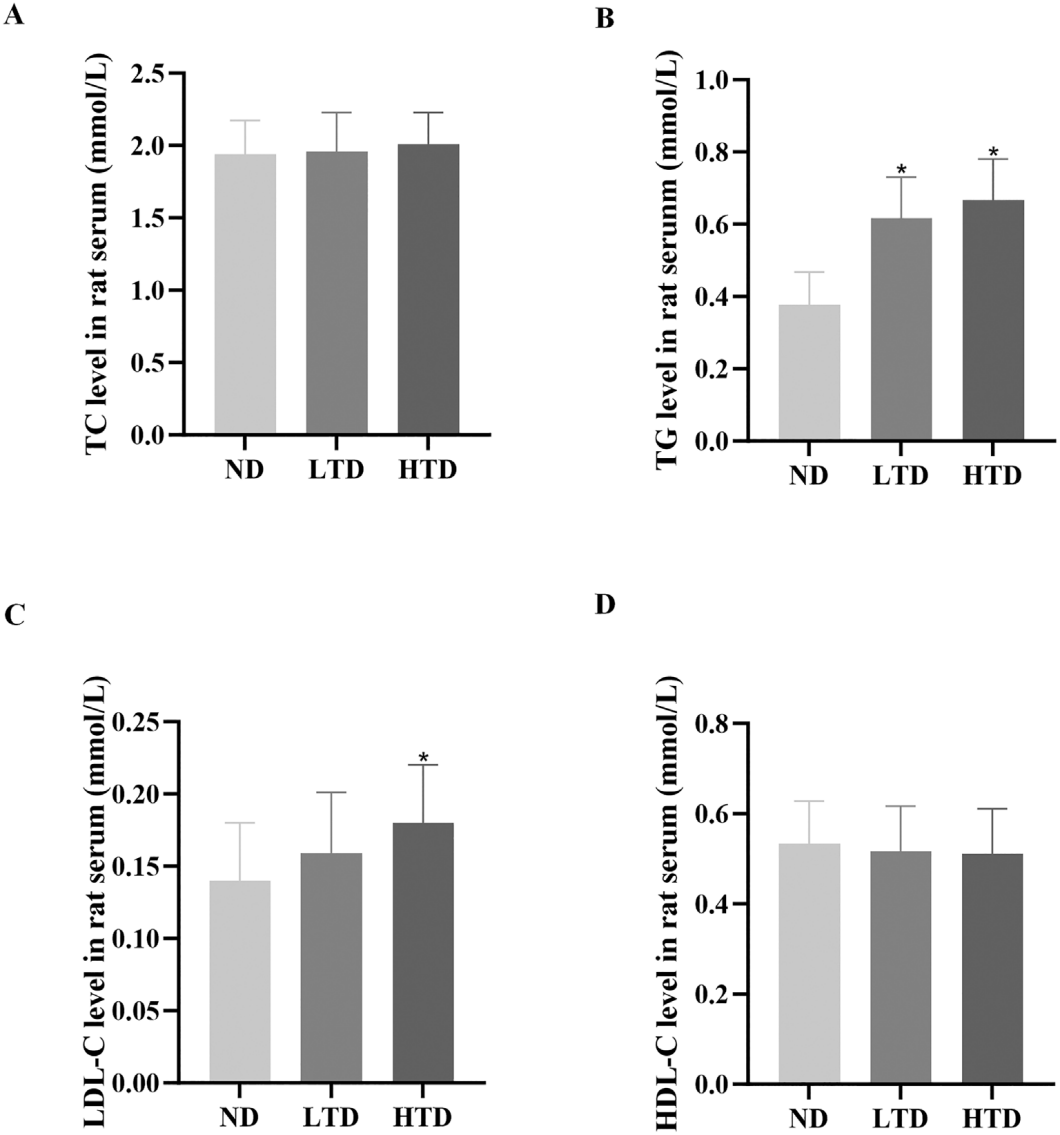
Effects of trans‐fatty acids on blood lipids among various groups. (A) Total cholesterol (TC) level in rat serum. (B) Triglyceride (TG) level in rat serum. (C) Low‐density lipoprotein cholesterol (LDL‐C) level in rat serum. (D) High‐density lipoprotein cholesterol (HDL‐C) level in rat serum. Results are shown as mean ± SD (*n* = 6). *p < 0.05 by contrast with ND group. ND, normal diet; LTD, 1% trans‐fatty acids diet; HTD, 4% trans‐fatty acids diet.

Serum lipid profiles were analyzed to assess the impact of TFA on lipid metabolism. Among the measured lipid indices, TG and LDL‐C levels exhibited statistically significant differences, whereas TC and HDL‐C levels remained comparable across groups (Figure 3). Compared with the control group, TFA‐treated groups demonstrated significantly elevated serum TG and LDL‐C levels (Figure 3B,C). In contrast, TC levels showed only a marginal increase, and HDL‐C levels displayed a slight decrease in the TFA‐treated groups relative to the control group (Figure 3A,D).

### 3.4 TFA Promotes Vascular Oxidative Damage in Rats

**FIGURE 4:**
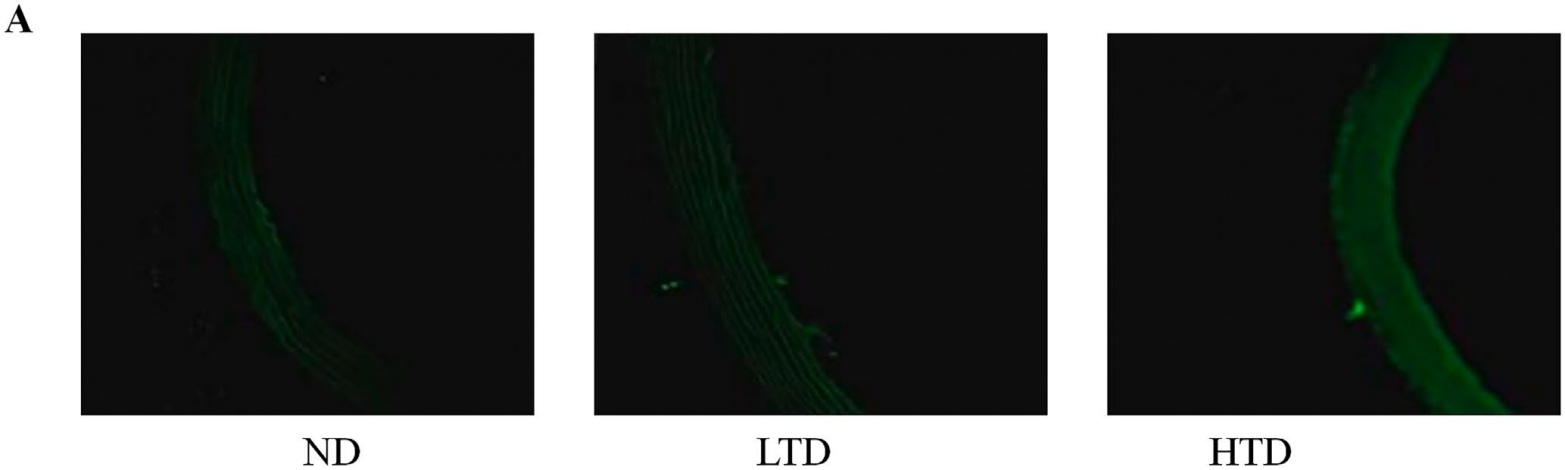
Effects of trans‐fatty acids on oxidative stress in blood vessels of rats on varied diets. (A) Expression of reactive oxygen species (ROS) in different dietary groups. ND, normal diet; LTD, 1% trans‐fatty acids diet; HTD, 4% trans‐fatty acids diet.

The unregulated generation of ROS is a key driver of oxidative stress, which plays a critical role in exacerbating vascular injury. Comparative analysis revealed that intracellular ROS levels were significantly elevated in a dose‐dependent manner with increasing TFA concentrations, as illustrated in Figure 4.

### 3.5 Effect of TFA on Rat Vascular Apoptosis

**FIGURE 5:**
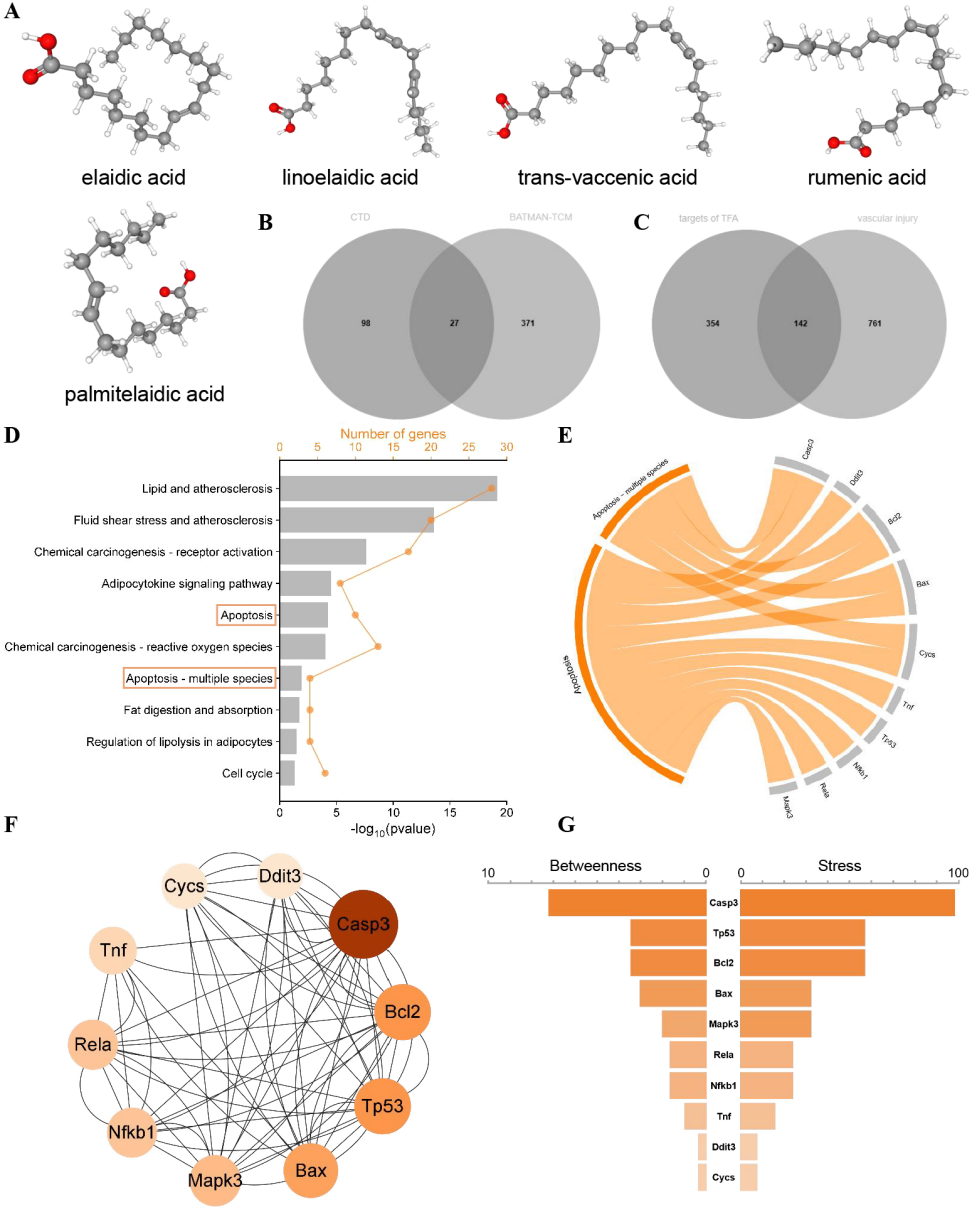
Computational pharmacology analysis of trans‐fatty acids (TFA) caused vascular injury. (A) Structures of the TFA utilized in the study. (B) Venn diagram showing the numbers of predicted TFA targets. (C) The overlapping targets of TFA and vascular injury. (D) Enrichment analysis for the intersection of TFA targets and vascular injury‐related targets. (E) Chord diagram representing the genes enriched in apoptosis‐related pathways. (F) Protein–protein interaction (PPI) network based on apoptosis‐associated proteins. (G) Screening of hub proteins.

**FIGURE 6:**
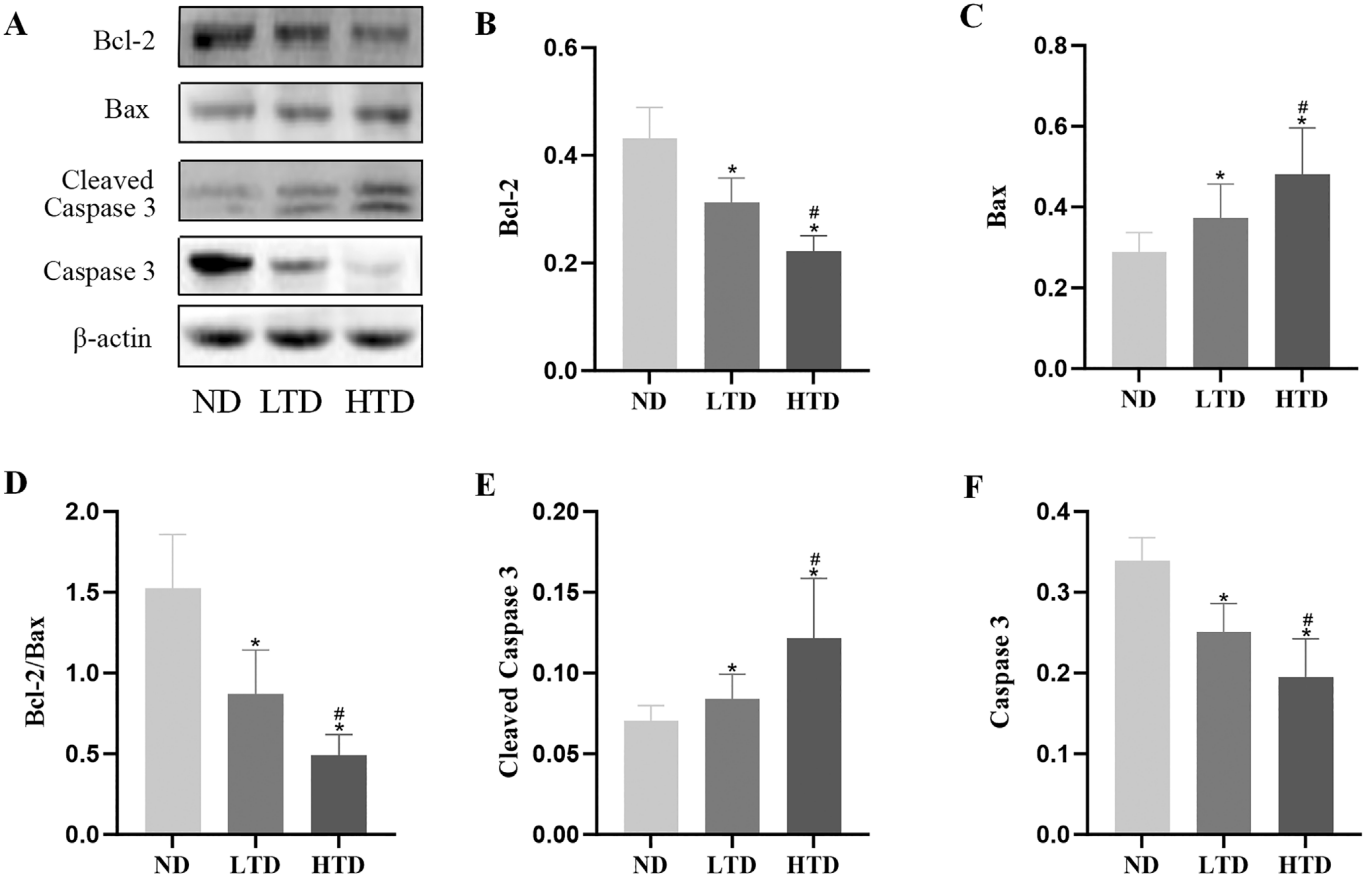
Effect of trans‐fatty acids on apoptosis of rat blood vessels. (A) Western blotting results for apoptosis‐associated proteins, including apoptosis regulator Bcl‐2, apoptosis regulator BAX (Bax), caspase 3, and cleaved‐caspase 3. (B–F) Semi‐quantitative analyses of protein expression. Results are shown as mean ± SD (*n* = 6). *p < 0.05 by contrast with ND group; ^#^ *p* < 0.05 by contrast with LTD group. ND, normal diet; LTD, 1% trans‐fatty acids diet; HTD, 4% trans‐fatty acids diet.

Through a systematic search and screening of the CTD, the BATMAN‐TCM, and the GeneCards database, 496 targets related to TFA and 903 targets associated with vascular injury were identified. The intersection of these two target sets yielded 142 candidate targets implicated in TFA‐induced vascular injury (Figure 5A–C). Kyoto Encyclopedia of Genes and Genomes enrichment analysis of these 142 genes revealed their biological functions, highlighting significant enrichment in pathways related to atherosclerosis, adipocyte regulation, and apoptosis (Figure 5D). Subsequently, a PPI network was constructed using the STRING database and visualized with the Cytoscape software, focusing on genes involved in the apoptosis pathway (Figure 5E,F). By applying two topological algorithms (Betweenness and Stress) via the CytoHubba plugin, the genes were ranked in descending order based on their scores. As shown in Figure 5G, TFA was predicted to significantly alter the expression of key apoptosis‐related genes, including Casp3, Bcl2, and Bax. To validate these findings, western blotting was performed to assess the expression levels of core apoptotic proteins. The results demonstrated a significant downregulation of Bcl2 and Casp3, accompanied by a marked upregulation of Bax and Cleaved Casp3 in TFA‐treated groups, confirming the activation of apoptotic signaling pathways (Figure 6A–F).

### 3.6 The Specific Mechanisms of Vascular Injury Induced by TFA in Rats

**FIGURE 7:**
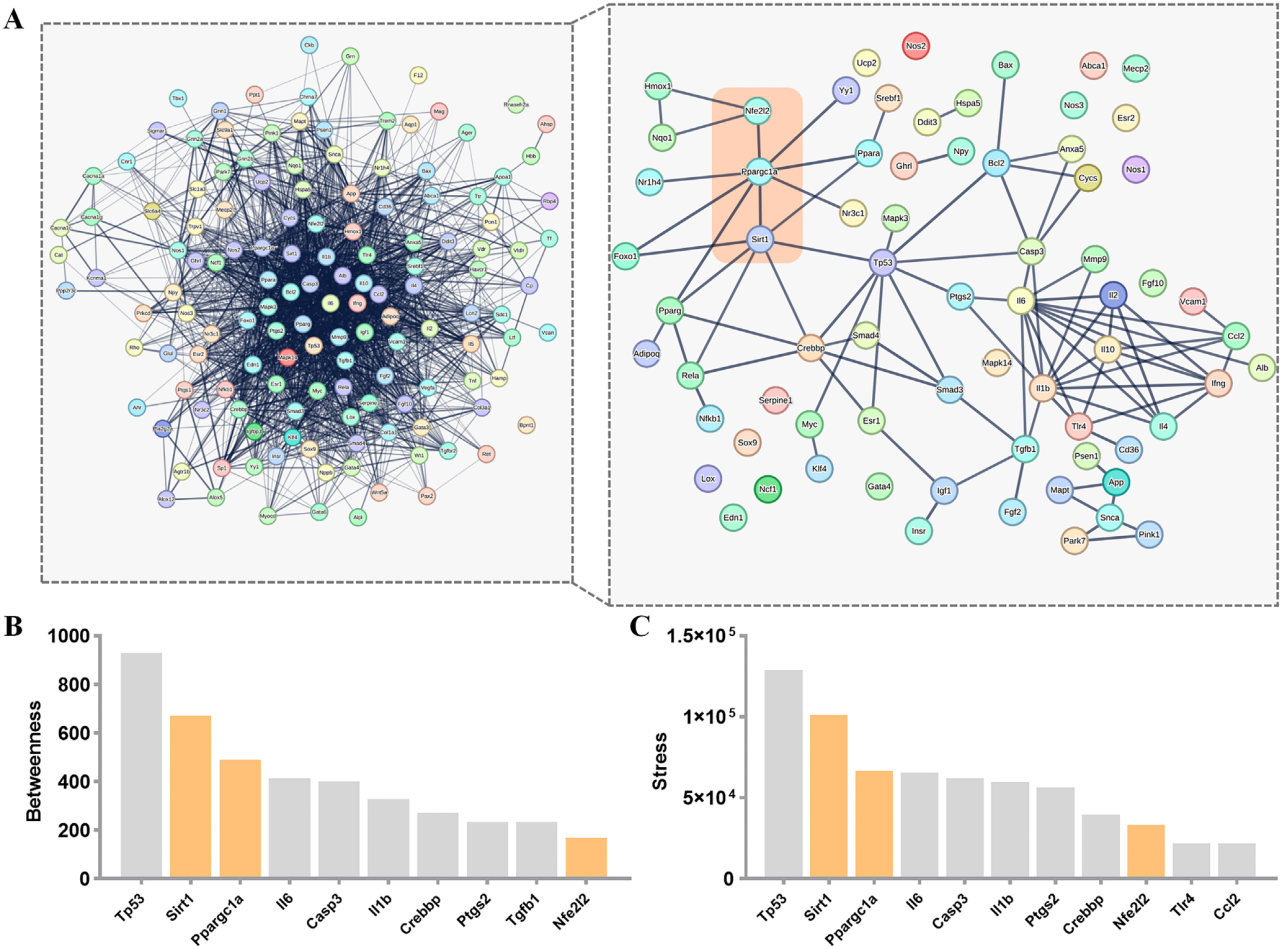
Protein–protein interaction (PPI) network based on the intersection of TFA targets and vascular injury‐related targets. (A) PPI network and its subnetwork based on Sirt1. (B and C) Screening of hub proteins based on the subnetwork.

**FIGURE 8:**
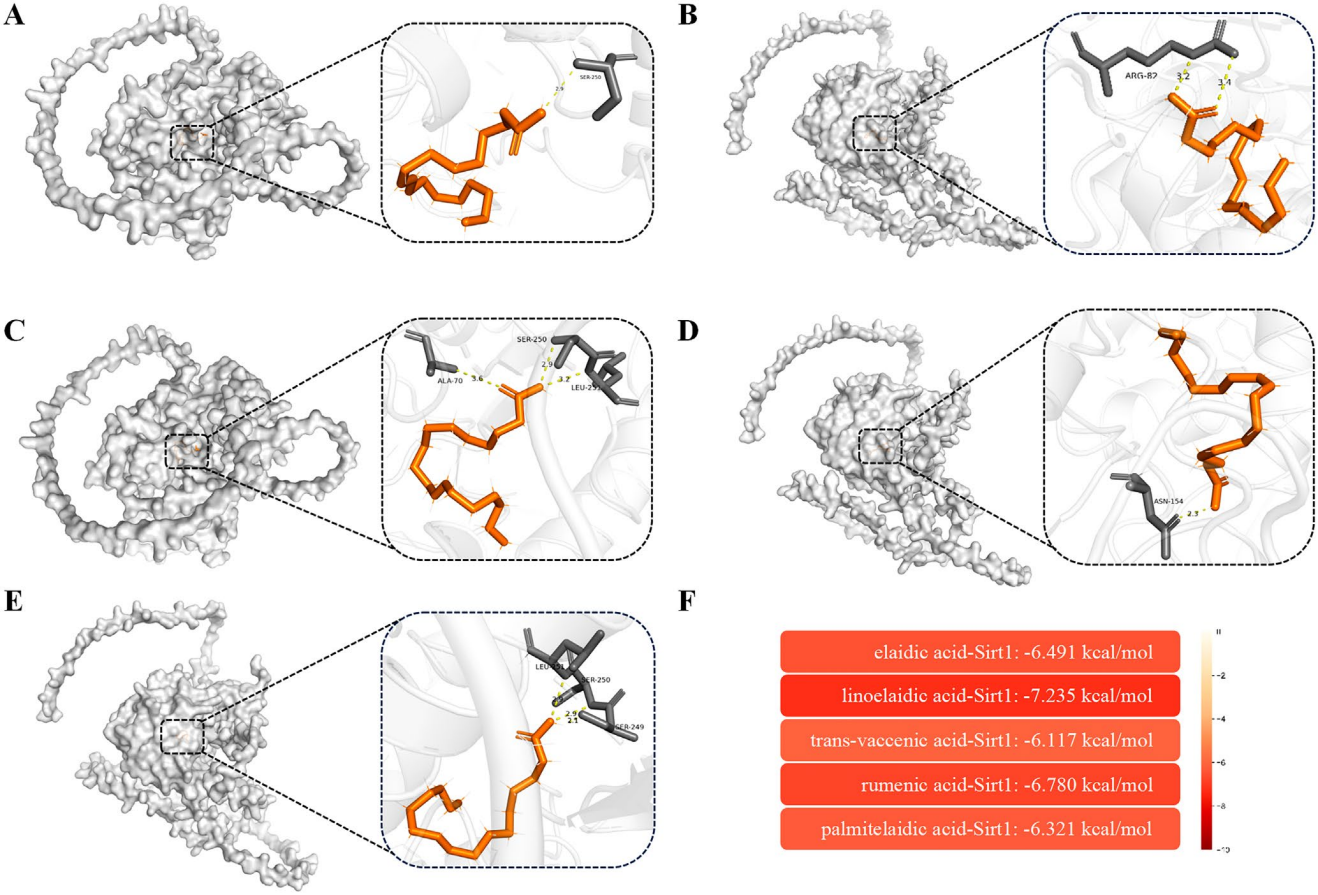
Molecular docking studies between TFA and Sirt1. (A–E) Docking results of elaidic acid–Sirt1, linoelaidic acid–Sirt1, *trans*‐vaccenic acid–Sirt1, rumenic acid–Sirt1, and palmitelaidic acid–Sirt1, respectively. (F) Heatmap of the docking results.

**FIGURE 9:**
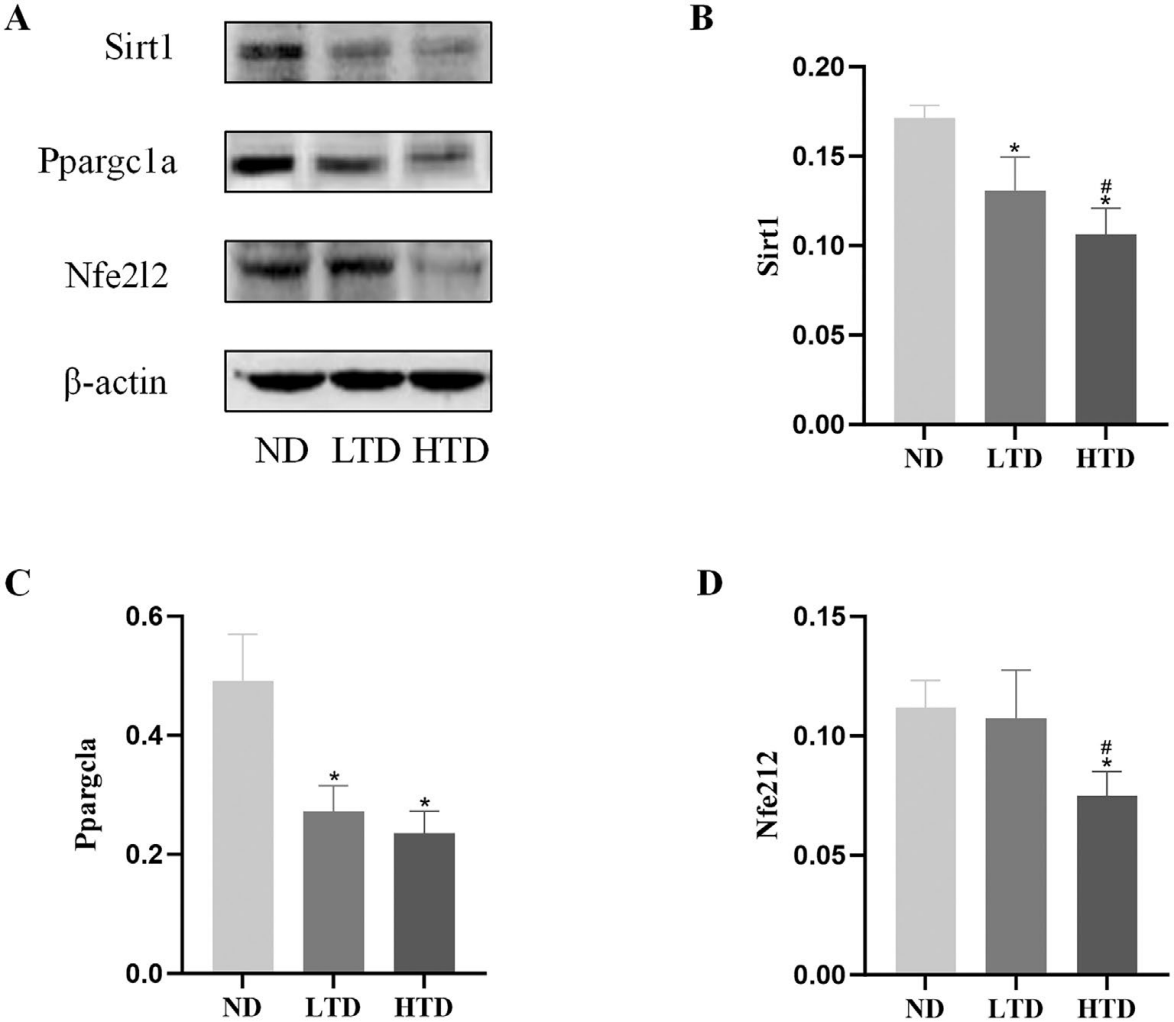
Effect of trans‐fatty acids on Sirt1/Ppargc1a/Nfe2l2 pathway of rat blood vessels. (A) Western blotting results of Silent information regulator 1 (Sirt1), Peroxisome proliferator‐activated receptor gamma coactivator 1‐alpha (Ppargc1a), and nuclear factor erythroid 2‐related factor 2 (Nfe2l2). (B−D) Semi‐quantitative analyses of protein expression. Results are shown as mean ± SD (*n* = 6). *p < 0.05 by contrast with ND group; ^#^ *p* < 0.05 by contrast with LTD group. ND, normal diet; LTD, 1% trans‐fatty acids diet; HTD, 4% trans‐fatty acids diet.

Next, we explored the specific mechanisms underlying TFA‐induced vascular injury. A PPI network was constructed using the intersection of TFA‐related targets and vascular injury‐associated targets. Previous studies have established that Sirt1 plays a pivotal role in apoptosis regulation (Zhang et al. 2016; Hacioglu 2022). Therefore, we extracted the Sirt1‐containing subnetwork for further analysis (Figure 7A). The subnetwork was screened using the betweenness and stress topological algorithms, and the top 10 target genes with the highest scores were identified (Figure 7B,C). Our analysis revealed that Sirt1, Ppargc1a, and Nfe2l2 emerged as core regulatory nodes. Molecular docking analysis demonstrated the binding sites and hydrogen bond lengths between TFA and Sirt1 (Figure 8A–E). The heatmap of docking energies (Figure 8F) revealed that all binding energies were below −5 kcal/mol, indicating strong binding affinities between TFA and Sirt1. Furthermore, western blotting results showed that the expression levels of Sirt1, Ppargc1a, and Nfe2l2 were significantly downregulated in TFA‐treated groups in a dose‐dependent manner (Figure 9A–E). Based on these findings, we propose that TFA induces vascular injury through the modulation of the Sirt1/Ppargc1a/Nfe2l2 signaling axis.

## 4 Discussion

TFA must be carefully considered in the safety assessment of foods and consumer products due to their detrimental effects on critical human physiological functions. Extensive epidemiological, clinical, and metabolic studies have established a strong correlation between dietary TFA intake and the risk factors as well as clinical outcomes associated with CVDs (Torrejón and Uauy 2011). Among these findings, a significant positive correlation has been consistently observed between TFA consumption and the incidence of CVDs (Laake et al. 2012). Clinical studies involving human subjects have demonstrated that this association is likely mediated by iTFA‐induced elevations in TC and LDL‐C levels, accompanied by a reduction in HDL‐C concentrations (Oteng and Kersten 2020). The intake of TFA has been identified as a significant health concern for both adult and pediatric populations. Globally, the levels of TFA vary substantially due to differences in dietary habits and the extent of iTFA used in processed food products (Craig‐Schmidt 2006). A multicenter study conducted by Jiang et al. across nine provinces in China revealed that the mean TFA intake among the study population increased from 0.25 to 0.53 g/d, marking a more than twofold rise over the past two decades (Jiang et al. 2020). In response, numerous countries have established regulations to limit TFA content in food products and have implemented strategies to reduce TFA consumption in the general population (Trattner et al. 2015; Kris‐Etherton et al. 2012). For the first time, our in vivo findings demonstrate that TFA induces mitochondrial damage by modulating the Sirt1/Ppargc1a/Nfe2l2 signaling axis, leading to elevated reactive ROS levels and subsequent apoptosis in vascular tissues.

After 12 weeks of dietary intervention, no significant vascular histological alterations were observed across all groups, as assessed by HE and Weigert staining. However, the severity of mitochondrial damage exhibited a positive correlation with the proportion of energy derived from TFA in the diets. Notably, the existing literature on TFA presents conflicting evidence regarding its role as either pro‐inflammatory or anti‐inflammatory (Oteng and Kersten 2020). Nevertheless, the observed mitochondrial damage may be attributed to the incorporation of the trans‐double bond structure of TFA into the inner mitochondrial membrane which subsequently disrupts the voltage‐dependent anion channel (VDAC) and reduces membrane fluidity (Tewari et al. 2015). Furthermore, our findings demonstrated that the TFA diet did not significantly affect physiological parameters, including body weight, food intake, and systolic and diastolic blood pressure, compared with the control group. Notably, the weekly food intake data revealed two transient reductions in consumption during Weeks 3–4 and Weeks 10–11 across all groups. Statistical analysis confirmed no significant differences between the TFA and control groups during these periods, suggesting that these fluctuations were not diet‐specific. We propose that these temporary declines reflect normal variability in feeding behavior, potentially associated with spontaneous changes in animal activity. Both animal and human studies suggest that long‐term TFA consumption may be associated with weight gain (Baylin et al. 2015). Limited but consistent evidence from epidemiological studies and primate models suggests that increased TFA consumption may contribute to modest weight gain. Additionally, data from a long‐term primate study indicate that TFA may exhibit a more pronounced adipogenic effect than cis‐monounsaturated fatty acids (Thompson et al. 2011). The absence of significant changes in body weight and food intake across different trans‐fatty acid groups in this study aligns with previous literature (Wanders et al. 2010). Although trans‐fatty acids have been proposed to possess potential obesity‐promoting properties, their effects may be modulated by multiple factors. Our animal study found no significant blood pressure alterations across TFA dosage groups, consistent with short‐term human trials (Desgagné et al. 2016). However, NHANES data link chronic TFA exposure to higher hypertension risk, suggesting our duration of intervention may fell below the hemodynamic effect threshold (Luan et al. 2024). A 12‐week intervention period may be insufficient to induce the metabolic changes required to significantly alter body weight or blood pressure. Longitudinal studies are better positioned to detect TFA‐induced obesity and hypertension.

Limited data are available on the metabolism of TFA. Previous studies have reported a tendency toward elevated TG levels associated with TFA consumption (Aronis et al. 2012). In addition to that, both iTFA and rTFA have been shown to increase cholesterol levels (Allen et al. 2015). Consistent with previous studies, our results also indicate an increase in TC and TG levels triggered by TFA. Similar to saturated fatty acids (SFA), TFA are linked to increased plasma LDL‐C concentrations. However, unlike SFA, TFA do not raise HDL‐C levels (Lichtenstein 2014). Studies have shown that participants consuming diets high in partially hydrogenated fats (TFA) exhibited lower HDL‐C levels compared with those consuming butter (rich in SFA) or soybean oil (rich in polyunsaturated fatty acids). In the present study, we observed a similar phenomenon, which may be explained by increased HDL apoA‐I catabolism, leading to a reduction in its pool size. Mitochondrial dysfunction can trigger excessive ROS accumulation, and prolonged ROS exposure exacerbates oxidative damage to mitochondria, creating a vicious cycle of mitochondrial impairment (Liu et al. 2021). In TFA‐treated groups, we observed severe mitochondrial damage in vascular tissues alongside ROS overproduction.

Network pharmacology, a concept introduced by Hopkins in 2007, provides a transformative framework for understanding the system‐level mechanisms of molecules or drugs (Li et al. 2020). This approach has emerged as a powerful tool for exploring molecular targets and elucidating the underlying mechanisms of bioactive compounds. The mechanism by which TFA induce vascular injury is highly complex, making it challenging to identify key insights solely through traditional experimental methods. However, network pharmacology combined with experimental validation offers a robust and practical solution to this problem. Through a comprehensive screening of multiple databases, we identified potential targets associated with TFA‐induced vascular injury and performed enrichment analysis on these targets. Among the KEGG pathway results, apoptosis‐related pathways were significantly enriched, drawing our attention. To date, whether TFA can directly induce apoptosis in vascular tissues remains unclear. Using topology analysis, we identified critical proteins in the apoptosis pathway, including Casp3, Bcl2, and Bax. Bcl2 and Bax are key regulators of apoptosis, with Bcl2 acting as an antiapoptotic protein and Bax as a pro‐apoptotic counterpart (Lin et al. 2021). Casp3, a well‐established effector protein, plays a central role in executing apoptosis (Karakaya et al. 2018). Western blotting analysis revealed significant activation of apoptosis, providing the first evidence that apoptosis is a central mechanism in TFA‐induced vascular injury.

To investigate the regulatory mechanisms of apoptosis, a subnetwork centered on Sirt1 was extracted. Using the aforementioned topological algorithms, Sirt1, Ppargc1a, and Nfe2l2 were identified as central proteins within the network that modulates apoptosis. Sirt1 is known to regulate apoptosis under pathophysiological conditions (Zhang et al. 2016). Previous studies have shown that SIRT1 inhibition impairs mitochondrial function and oxidative phosphorylation, thereby promoting follicular apoptosis, whereas SIRT1 overexpression restores mitochondrial function and reduces follicular atresia (Guo et al. 2022). In the current study, western blotting analysis revealed that the marked downregulation of Sirt1 is associated with TFA‐induced vascular apoptosis, highlighting a novel role of Sirt1 in this process. Furthermore, we observed that Sirt1 inhibition was correlated with Ppargc1a downregulation. Ppargc1a, a key regulator of mitochondrial biogenesis, plays a critical role in mitochondrial renewal (Zhong et al. 2019). Suppression of Ppargc1a expression reduces mitochondrial biogenesis and leads to the accumulation of damaged mitochondria (Pousa et al. 2021). As a known target of Sirt1, the upregulation of Ppargc1a may explain the potential protective role of Sirt1 in maintaining mitochondrial homeostasis (Maizel et al. 2014). Nfe2l2, a transcriptional factor, upregulates genes involved in mitochondrial integrity, oxidative phosphorylation efficiency, mitochondrial biogenesis, and DNA repair (Zhang et al. 2018). Collectively, these findings suggest that TFA promotes vascular apoptosis by targeting the Sirt1/Ppargc1a/Nfe2l2 signaling axis.

In summary, our findings demonstrate that TFA induces mitochondrial damage, resulting in elevated ROS levels and subsequent vascular apoptosis via the Sirt1/Ppargc1a/Nfe2l2 signaling axis. However, our study did not analyze the specific composition of TFA, and further research is needed to investigate the impact of TFA with different structures.

## Author Contributions

**Huanting Pei:** data curation (equal), methodology (equal), writing – original draft (equal). **Ran Li:** formal analysis (equal), methodology (equal). **Yadong Zhang:** investigation (equal), methodology (equal). **Xiaoya Sun:** data curation (equal). **Siqi Zhu:** data curation (equal). **Simeng Qiao:** conceptualization (equal). **Chongyue Zhang:** conceptualization (equal). **Xiaolong Zhang:** conceptualization (equal). **Jingyi Ren:** investigation (equal), methodology (equal). **Yuxia Ma:** funding acquisition (equal), writing – review and editing (equal).

## Conflicts of Interest

The authors declare no conflicts of interest.

## Data Availability

The data generated during the current study are available from the corresponding author upon reasonable request.
